# Data-Driven Monitoring in Community Based Management of Children With Severely Acute Malnutrition (SAM) Using Psychometric Techniques: An Operational Framework

**DOI:** 10.7759/cureus.18589

**Published:** 2021-10-07

**Authors:** Ankur Joshi, Abhijit P Pakhare, Sivaja K Nair, Revadi G, Manoj Chouhan, Deepak Pandey, Arun M Kokane

**Affiliations:** 1 Epidemiology and Public Health, All India Institute of Medical Sciences, Bhopal, IND; 2 Community and Family Medicine, All India Institute of Medical Sciences, Bhopal, IND

**Keywords:** mortality, rehabilitation, supervision, monitoring, rasch analysis, severe acute malnutrition

## Abstract

Background

The success of the Community Based Management of Severe Malnutrition (CSAM) programme largely depends on the knowledge and skills of Front-Line Workers (FLWs). A robust supportive supervision system in CSAM should be tailored to individualistic learning needs by distinguishing the FLWs as per their ability and simultaneously identifying the task domains to be emphasized more in supervisory visits. This paper details the ability assessment strategy developed and employed in the selected geographical locations in Madhya Pradesh (Central India) among the 197 Anganwadi workers (FLWs involved in CSAM implementation).

Methodology

A 25 items tool was developed based on an analytical construct for ability estimation through Rasch Analysis (RA). RA models the probability of right/wrong answers as a function of a person (participants) and item (questions) parameters and calculates the item difficulty in relation to personability on the same unidimensional linear scale. Suitable visualization like item characteristic curve (ICC), person item map (PIM) and quadratic allocation were plotted in RA. The data fitting to the Rasch model (Rasch diagnostic) was tested by numeric (Anderson LR and Wald test) and graphical methods.

Results

The item easiness parameter (β) value related to Diarrhoeal assessment was lowest (-2.32, -2.91 to -1.73) and related to peer assessment meaningful action (2.009, 1.669- 2.348)) was highest (most difficult). Anderson LR test (LR=31.32, df=24, p=0.079) showed the absence of global outliers. Quadrant analysis using the permutations of ability score and adjusted burden of malnutrition further mapped 41/197 (20.8%) FLWs to low ability -high burden quadrant and 44/197(25%) as low ability low burden quadrant.

Conclusion

Rasch assessment may address the innate challenges to maintain homogeneity, discrimination capacity and linearity in a raw score-based measurement construct. The monitoring strategy developed on this thus may offer a judicious, pragmatic and thematic approach to supportive supervision in the CSAM program.

## Introduction

Severely Acute Malnutrition (SAM) is a condition characterized by extremely low weight for height, muscle wasting and nutritional oedema. It is closely associated with high mortality and morbidity rates among children under five [1]. A child with SAM or MAM (Moderate Acute Malnutrition) has more likelihood of mortality than a well-nourished child if not intervened promptly [2]. Until the first decade of this century, all such children were primarily approached through facility-based care through a specifically designed intervention centre known as Nutritional Rehabilitation Center -NRCs. This facility-based care consisted of medical management of complications (such as correction of hypoglycaemia, hypothermia, shock, electrolyte imbalance), feeding interventions (starter and rehabilitative diet, food chart) and educational interventions (involving mother in food preparation, care of a child after discharge). This is also known as facility-based management of SAM children (FSAM). However, this strategy faced several challenges such as high operational and opportunity costs (for both systems and parents of SAM children), low coverage; longer stays leading to overcrowded NRCs, and cross-contamination [3-5].

Moreover, multiple studies have stated that only 10-20% of the SAM children developed complications requiring hospitalized care. In contrast, the rest could be treated at the community level with a package of services (medicines, nutritional supplements, nutrient-dense foods and weekly tracking) [3,6]. To address this gap, Community Based Management of SAM children (CSAM) came into existence [7-9]. It is also known as Community based Management of Acute Malnutrition (CMAM) globally.

CSAM offers an opportunity for longitudinal tracking of the child by an intersectoral peripheral designated team at their doorsteps to monitor their nutritional status continuously. This also includes the initial and follow up assessment for medical complications and subsequent referral to the health facility. Several processes beginning from initial screening, classification, management and subsequent follow-ups of the beneficiaries in CSAM are mostly performed by the peripheral Front-Line Worker (FLW) team constituted chiefly by Anganwadi Worker (AWW) and Auxiliary Nurse Midwife (ANM). Thus, the program's effectiveness in principle depends on the robust ability of these FLWs to understand, apply and make decisions as per programmatic guidelines.

Thus, this program's monitoring and supervision strategies must be sensitive enough to capture the extent of the ability of FLWs about the conceptual understating and translating deliverables in CSAM strategy, without considering this aspect negates the principles on which they were built. The ability estimation is an abstract and latent construct in the Psychometric paradigm. Generally, the measurement of this construct is attempted by some manifested variables (henceforth referred to as items), which are presumed to measure the unique fragment of that construct. The significant sum of these items is perceived as ability. This seemingly easy concept of ability measurement is a little tricky on the operational plane, especially in social sciences /psychometrics. The raw scores derived by this summation may ignore the equidistance and linearity of items on difficulty level [10]. Thus these items may not discriminate the high from low achievers in the true sense. Neither offers an insight to the evaluator whether he measures a homogeneous construct accurately only [11-13]. There are multiple studies on the assessment of skills and capacities of the frontline workers; a few regarding programmes about the management of malnutrition and few others on generic public health programmes [14-16]. However, most of them estimate the ability by raw score without any transformation that neither considers the item difficulty and associated discrimination capacity nor addresses the equidistance distribution of items and participants on a linear scale. Translating it from a programmatic stance, the differential capacities of FLWs on different domains of tasks may not be explicitly addressed through them.

Rasch Analysis (RA) offers an alternate strategy to estimate the ability is offered by Rasch Analysis (RA), which considers the homogeneity, discrimination capacity, and linearity of items [17]. This is a mathematical modelling technique routed in logarithmic transformation. It attempts to achieve a conjoint (person-item measurement on the same linear scale) additivity (equidistance linearity). In contrast, the homogeneity of the tool is simultaneously attempted to maximize by reducing items (poor discriminator, misclassifies etc.)[18]. It presumes that the probability of a correct response on an item is the product of calibrated item difficulty and calibrated person ability measured on a logarithmic scale. A further description is of this scale is given in the supplementary file.

We developed a data-driven monitoring strategy within the context above that considered malnutrition magnitude concerning ability (measured by RA) estimation. The objectives for this strategy development were to identify the AWCs requiring frequent supportive supervisory visits and to identify the programmatic components to be addressed during the visit.

This article was previously posted to the medRxiv preprint server in June 2021.

## Materials and methods

This study was designed by the CSAM unit of the Regional Center of Excellence for Nutritional Rehabilitation and Resource Training (RCoENRRT), Madhya Pradesh. The RCoE was established under the aegis of AIIMS Bhopal, an apex teaching tertiary care hospital in Central India. The RCoE has adopted a sub-district development block (Babai) from the district of Hoshangabad for technical facilitation, execution and supportive supervision of the CSAM program. The methodology section is further subdivided into three different subsections for ease of understanding.

A1 Tool development- 

The process of tool development started in September 2019. The first step in tool development was identifying the theoretical construct from literature review and in-house discussions among investigators. This process led to the identification of three domains, namely performance, adaptability and stability, that could be further contextualized for CSAM management [19,20]. The initial desk review was followed by four field visits and engagement with the field experts to refine identifying the subdomains specific to the context. The identified subdomain within the analytical framework is detailed in Table 1.

**Table 1:** Analytical construct defining domains and subdomains for the study. CSAM- Community based management of severe acute malnutrition

Domains	Subdomains
Performance Operational identification: encompassing the level of knowledge and skills to transform this knowledge into deliverable actions	Understanding about the CSAM programme implementation Knowledge about the processes involved. Skills in anthropometry Skills in classification of the children
Adaptability Operational identification: capacity to perform in the course of adverse events and changing needs of the community	Capacity to deliver services as per the differential need of the community. Capacity to understand the social and cultural premises of malnutrition.
Stability Operational identification: degree to which an individual can decrease volatility of performance through adaptation of good practices and norms	Preparedness Skill for risk mitigation

After identifying the subdomains, the research team developed the items and transected the items as a vital, essential and desirable matrix for each subdomain. We had developed 36 items initially based on the subdomains. The items were put to discussion with the research and field experts to decide on the validity of the content, structure of the contents, and the length of the questionnaire. The items were proposed to be closed-ended with multiple choices. This review of the initial pool of items reduced the number of questions to 30 once the redundant and repetitive questions were deleted from the list. A few of the questions were changed as per the expert opinion considering its contextual applicability. These questions were further translated into the local language with the help of a field expert proficient in the vernacular language. The translated items were put for pre-test with seven research participants to check for issues in the language, tone, structure, and design of a questionnaire. Incorporating the suggestions from the research participants and the identified need, changes were made to the questions, and the number of items was reduced to 27. A round of pilot tests was undertaken to assess the consistency and reception of questions. Relevant changes were made to the pool of items, and the item numbers were finalized as 25. Another round of pilot tests was undertaken with the items before finalizing the tool to assess clarity and ease of understanding.

Information on the burden of malnutrition for each Anganwadi centre was retrieved from a monthly report submitted at the block office. The number of children with Severe Acute Malnutrition (SAM-weight for height Z-score <-3) and Moderate Acute Malnutrition (MAM-weight for height Z-score <-2) was noted.

A2 -Tool institution

The pre-tested and piloted tool was ready to use by October 2019. It was a community based cross-sectional psychometric health system research. As planned and communicated earlier, the Anganwadi workers (AWW) of Babai block were called for a block-level review meeting on the CSAM programme to the Block Panchayat through the Child Development Project Officer (CDPO) on 11 October 2019. It was decided to conduct the Rasch assessment prior to the review meeting. Out of 217 AWWs in the Babai block, 197 AWWs were present for the exercise. The pre-printed questionnaires were handed out to the AWWs. None of the questions was explained or read out to the research participants even when in doubt, since an individual level of understanding of the question was the mandate for the assessment. It was also communicated with the participants not to have any discussions on the questions. All possible measures, including a time frame of 30 minutes for completing the questionnaire, were set to avoid potential bias in the execution process.

A3 Analysis and model creation

The data was entered in MS Excel in wide data format and was checked for duplication, missing values, outliers and redundancies. The adjusted total burden of malnutrition was calculated for each Anganwadi Centre (AWC- Geo-administrative unit for AWW) by assigning half of the weight (0.50) to a MAM child compared to a SAM child. The analysis comprised 25 items and 197 respondants i.e. Anganwadi Workers (AWWs). The analysis was performed in base-R and r-packages available in open domains. For each item, difficulty parameters with confidence intervals were obtained. Item Characteristic Curves (ICCs) were drawn for each domain item which showed the plot between the probability of correct response for an item on the y-axis and the underlying ability for the domain item on the x-axis. The purpose was to have a visual idea of the discrimination capacity and difficulty of the items clubbed under the domain construct.

A person-item map was plotted through the eRM package. This map displayed the range and position of the item (lower panel) and person parameters (upper panel) on the same axis of latent dimension.

The Rasch model was initially created using responses of 197 participants for 25 items. The data fitting to the Rasch model (Rasch diagnostic) was tested by numeric (Anderson LR and Wald test) and graphical methods. The reason behind choosing the two tests was to look at the model both from a global (all items simultaneously) and an individualistic item wise perspective. The Anderson LR statistic checked for the item bias (differential item functioning globally), while the Wald test looked for the individual item invariance by dividing the sample into two groups. The visual diagnostic was performed by using the Goodness-of-Fit plot. Here, underlying assumptions of homogeneity (with confidence bend) was visualised by comparing the beta scores of two groups split by median score as a cut-off. Rasch model was scrutinised to check for deterministic patterns by infit MSQ value. All the diagnostics were run on eRM R-package. 

**Figure 1:**
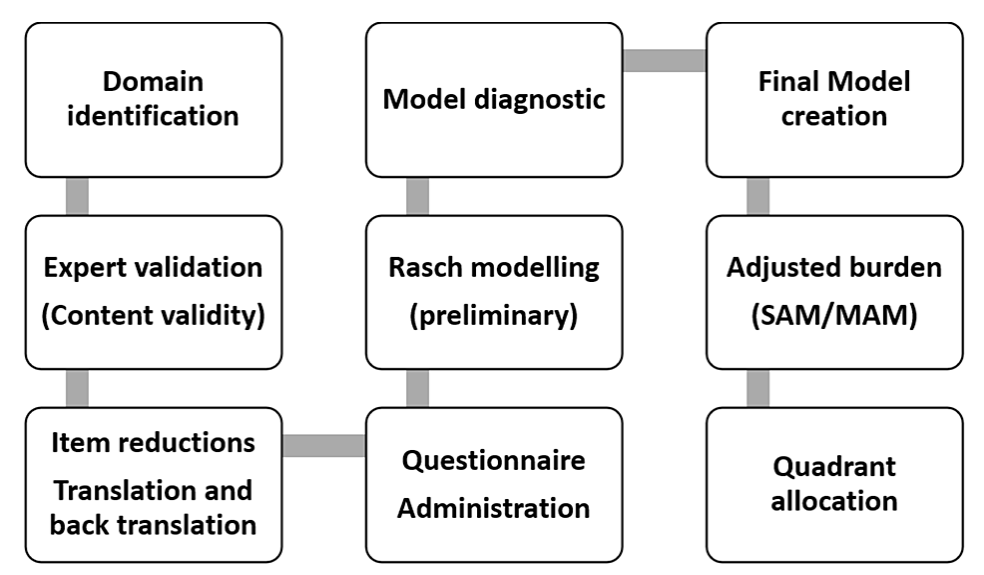
From Construct identification to model creation

A scatter diagram was drawn with the ggplot2 package. Each co-ordinate represented the adjusted burden of Malnutrition in AWC at the x-axis and Rasch value scored by corresponding AWW at the y-axis. The malnutrition burden in a centre was thought off in terms of the total weighted number of SAM and MAM children enrolled in the centre at the time of tool administration. A MAM child was given half of the weight compared to a SAM child for calculating the burden, which ensured the relatively high contribution of SAM into the total burden. This scatter plot was further divided into four quadrants by placing a horizontal and vertical line intersecting at the y and x-axis. These intersecting lines represented the median values of Rasch score (horizontal line) and adjusted burden of malnutrition (vertical line). This exercise led to the formation of four quadrants- Q1 (right lower)-low ability high burden, Q2(left lower)-low ability, low burden, Q3(left upper)-high ability, high burden and Q4(right upper)- high ability, low burden. Figure 1 illustrates the process from construct identification to model creation in a sequential manner.

Ethics issues

The Institutional Human Ethics Committee of AIIMS Bhopal reviewed and approved the study protocol. Committee also granted a waiver of written informed consent. All participants were provided with an information sheet that had details about objectives and procedures. It was assured that performance in the assessment did not have any unfavourable effect on their regular working. There are no competing interests. It is a funded project by UNICEF, Madhya Pradesh.

## Results

**Figure 2:**
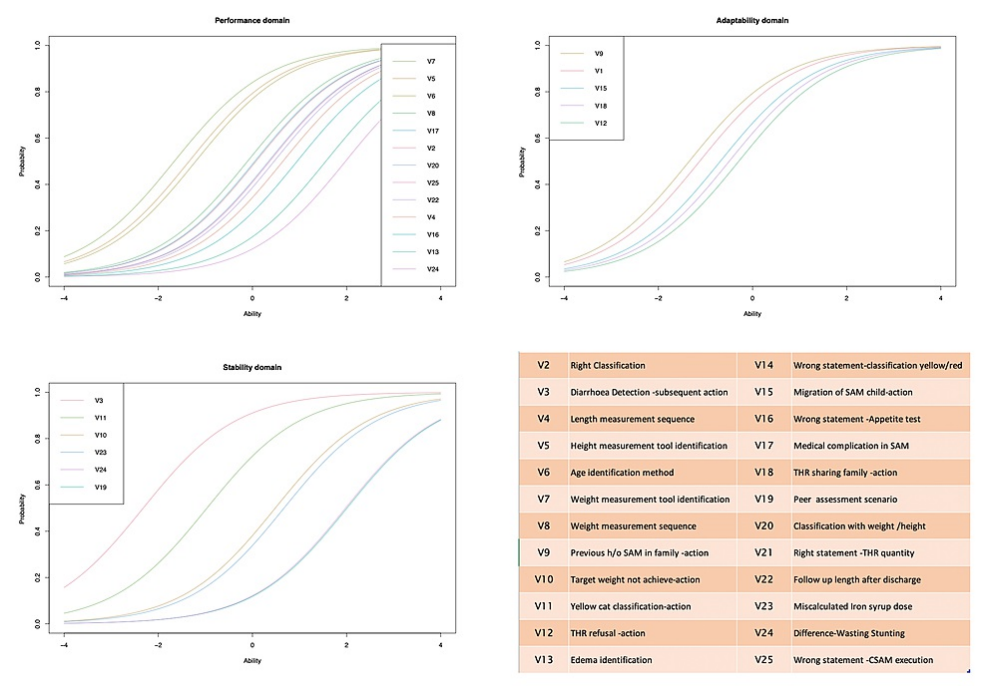
Item characteristic curve showing the monotonic homogeneity of ability on x-axis and probability to resolve at y-axis across the 3 domains of inquiry

Out of 217 AWWs in the Babai block, 197 completed the assessment. The average age of participants was 39.8 years, and the average duration of experience as AWW was 11.4 years. The item related to diarrhoeal assessment was found to be as easiest (-2.32, -2.91 to -1.73). Item related to peer assessment consequential action (2.009, 1.669- 2.348) was most difficult on item difficulty parameters. The Item Characteristic Curve (ICC) is shown in Figure 2, in which items were listed as per their domain membership with mean item difficulty scores. The items displayed in Figure 2 relate to 'stability' domains that were perceived easy by AWWs and had less discriminatory ability between low and high ability AWWs. On the other hand, items related to adaptability and performance had a higher discriminatory capacity and were perceived as difficult by AWWs.

**Figure 3:**
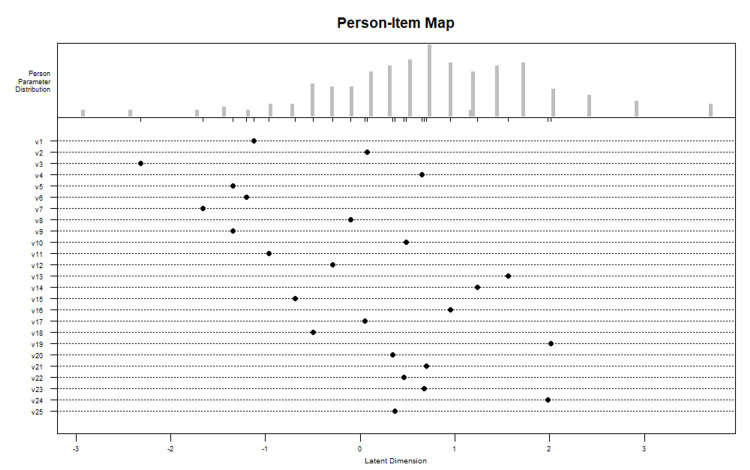
Person Item Map mapping the items and person along the same linear latent ability construct.

Person item maps in Figure 3 show that items were distributed along with the whole range of latent dimensions, thus sensitive enough to capture the whole of the latent construct. However, this sensitivity was lower at very high ability (to the right upper end). Item no 4,21 and 23 mapped the maximum number of participants.

The purpose of the Rasch analysis was to assess the difficulty level apart from the discriminatory capacity of items that ICC and the person item map showed. The difficulty level was assessed at item and person levels with the same latent dimension. Next, Table 2 shows the Item difficulty point estimates with their 95% confidence level. The items having beta estimate values with a negative sign connote the relatively easy items compared to items having beta estimates with a positive value

**Table 2:** Item difficulty parameters showing point estimates with 95% CI.

Item Code	Item Description	Beta estimate	Lower CI	Upper CI
V2	Right Classification	0.076	-0.23	0.382
V3	Diarrhoea Detection -subsequent action	-2.319	-2.906	-1.732
V4	Length measurement sequence	0.648	0.353	0.944
V5	Height measurement tool identification	-1.344	-1.761	-0.928
V6	Age identification method	-1.205	-1.605	-0.805
V7	Weight measurement tool identification	-1.659	-2.12	-1.197
V8	Weight measurement sequence	-0.103	-0.416	0.21
V9	Previous h/o SAM in family -action	-1.344	-1.761	-0.928
V10	Target weight not achieve-action	0.486	0.189	0.782
V11	Yellow cat classification-action	-0.965	-1.338	-0.591
V12	THR refusal -action	-0.292	-0.614	0.03
V13	Oedema identification	1.56	1.246	1.873
V14	Wrong statement-classification yellow/red	1.234	0.932	1.537
V15	Migration of SAM child-action	-0.684	-1.032	-0.335
V16	Wrong statement -Appetite test	0.95	0.653	1.247
V17	Medical complication in SAM	0.051	-0.256	0.358
V18	THR sharing family -action	-0.495	-0.83	-0.16
V19	Peer assessment scenario	2.009	1.669	2.348
V20	Classification with weight /height	0.344	0.045	0.643
V21	Right statement -THR quantity	0.695	0.399	0.99
V22	Follow up length after discharge	0.462	0.165	0.759
V23	Miscalculated Iron syrup dose	0.672	0.376	0.967
V24	Difference-Wasting Stunting	1.978	1.641	2.316
V25	Wrong statement -CSAM execution	0.368	0.069	0.666

Similarly, person parameters were also identified for 197 AWWs on the same latent dimension as of item parameter.

The descriptive analysis was followed by model diagnostic. Anderson LR test (LR=31.32, df=24, p=0.079) was applied to check the presence of global outliers, which showed the absence of such outliers. Similarly, local outliers were checked through Wald statistics. Twenty-one out of 25 items had a p-value more than the threshold, while four items (item-12,19,24 and 25) had p values less than the threshold (<0.05). However, these items were retained in the final model as they were in alignment with the construct.

**Figure 4:**
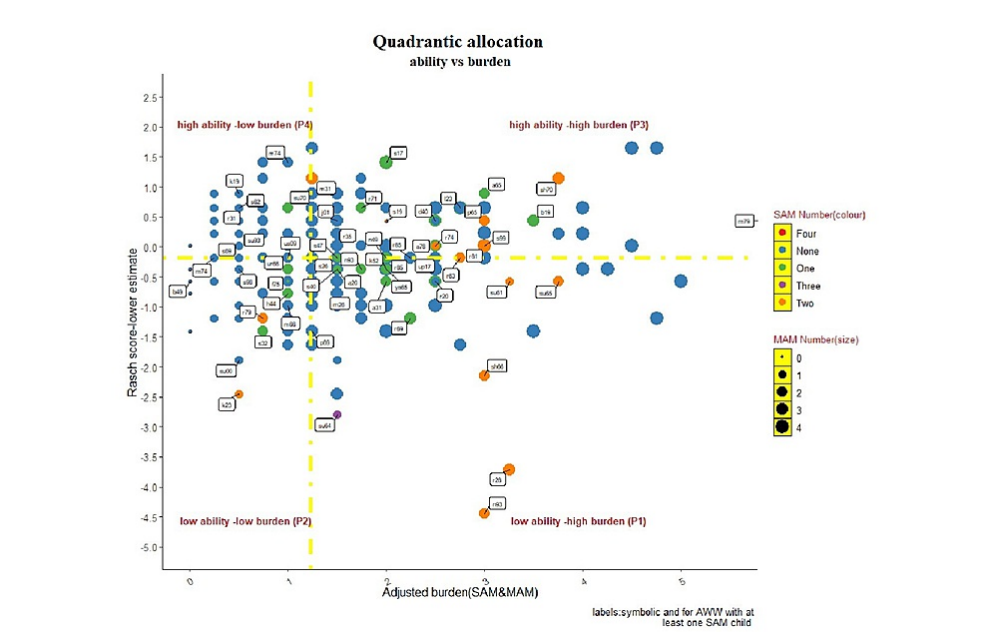
Quadratic allocation of AWWs as per ability (determined through Rasch) and adjusted burden of malnutrition. The yellow dashed line intersects the x and y-axis at their median value. The SAM number was colour coded, while the differential size of the dots showed MAM numbers. AWW- Anganwadi Worker; SAM- Severely Acute Malnutrition; MAM- Moderate Acute Malnutrition

The Rasch analysis and model diagnostic was followed by allocation of all the AWWs into one of the four quadrants created as under-low ability -high burden (P1- 41 AWWs), low ability-low burden(P2- 44 AWWs), high ability-high burden (P3- 37AWWSs) and high ability -low burden(P4- 75 AWWSs). This allocation of AWW as per the ability in CSAM domains and adjusted burden of the malnutrition (differential weightage of MAM and SAM child ) was shown through a visualization (Figure 4). All the AWWs were categorized into one of the four quadrants. About 38% of AWWs had high ability and low burden (low priority for monitoring with an inherent assumption of correct reporting as a function of ability). However, about 1 in 5 AWWs had a low ability and reported a high burden of SAM (P1), and about 1in 4 AWWs had a low ability and low reported burden of SAM (P2). These P1 and P2 segments were thought of as a priority segment for supportive supervision. This conceptual priority assignment was operationalized in 2 ways- enhanced relative allocation of monitoring visits to P1 and P2 segments and identification of difficult items from poor performing domains (by looking at item easiness parameters ) with individualistic /group based capacity building. 

## Discussion

There has been a global shift in the way in which the process of supervision was perceived, from authoritative mechanistic supervision ("do as I say") to supportive facilitatory ("do as we agree upon") supervision [21,22]. To embark on this paradigm, the supervisor should have a fair orientation of the strength and scope of improvement of the supervisee in his individualistic capacity before and during interaction in an objective manner. In practice, it is often difficult to achieve due to previous cumulative predispositions and the absence of tangible performance measures which have the factual and accurate construct of performance discrimination [23,24]. Rasch modelling may attempt to overcome both concerns as it assigns objectivity to the whole evaluation framework and has an innate discriminant capacity for ability estimations [18]. In addition, any ability estimation has the meaning only if it can be translated into the extent of task accomplishment. With the argument, any monitoring strategy that simultaneously contemplates ability and an intended task may offer more vital cues for actions than one that focuses only on ability or intended task [23-25].

The aptness of any measurement depends on the notion of a single construct (without contamination) equidistance and linearity in measurement in general. This is relatively easy to achieve in physical measurements by concatenation. In the world of social science/psychometrics, this measurement allocates the numeral to events as per some pre-defined rubrics. The accumulation of these numbers is perceived as magnitude for that event [26]. This simpler and linear calculation of the magnitude of an event will be problematic in social sciences since the observations do not fall equidistant from each other, and the positioning of a score has deeper meanings than its numerical value. A linear assessment method might offer a flawed interpretation of the results and understanding the events in place [18].

Several supervisory insights may be derived through this strategy- At first, in the high burden and low ability quadrant, we will know whether this burden is an actual burden or an anthropometric classification error on the fieldworker side? This question becomes more relevant in the presence of high discordance in MAM and SAM numbers. Although the discordance between SAM and MAM is reported by other population-based surveys and plausible explanations for the same is offered, the transition from MAM to SAM should be perceived from the CAS (Complex Adaptive System) perspective where system outputs are the non-linear sums of underlying processes, and this transition should be seen on a continuous pathophysiological spectrum from normal to SAM child and not as disconnected dichotomized entity. In the Quadrant plot, the absolute SAM burden corresponds to the marker's colour, thus adding to another dimension in the plot. Second, the low burden and low ability quadrant also conveys the same notion as mentioned above; whether are we missing some eligible SAM children here? The peripheral staff serves as 'gate-keeper of CSAM management ecosystem through identification of SAM/MAM children. Thus, the delayed entry or missing cases may adversely affect the program performance, leading to preventable child mortality. Third, the high ability quadrants also have a high and low burden of the problem. These quadrants may serve as benchmarks for estimating the true burden but with some caveats. The cut-offs are derived from the median scores; thus, these quadrants are the outcome of rank order arrangement in two dimensions.

Although this argument assigns more credibility to allocate the 'grey-zone markers into respective quadrants, yet farthest points at the quadrant plots should be interpreted with some caution. The standard error for the Rasch score is maximum at the extreme ends and minimum at the middle, which is the opposite of raw scores. Ability estimation accordingly may have more reliability at the middle compared to extreme ends [27]. This inherent limitation of Rasch transformation may not substantially affect the person-item map; the item density is more at the middle and none at the end. Finally, the difficulty scores having agreements between predicted difficulties by investigators and actual difficulties are the definite domains in which capacity building is required. The RCoE has organized the refresher training for AWWs targeting specifically these domains both as groups and on a one-to-one basis during monitoring visits. Apart from the innate weakness of lesser discrimination at both ends of the ability, the results should be interpreted with a caveat that the process of transformation of ability into Rasch score preserves order from a unidimensional perspective only while real-world ability might be multidimensional ( socio-environmental aspects, for instance ) in nature which is not accounted for while estimating the scores. However, all the responders belonged to the same socio-geographical strata and had comparable formal qualifications. Also, some participants may find it difficult to recall the correct numeric options in the presence of distractors.

Implications for policy and practice

**Figure 5:**
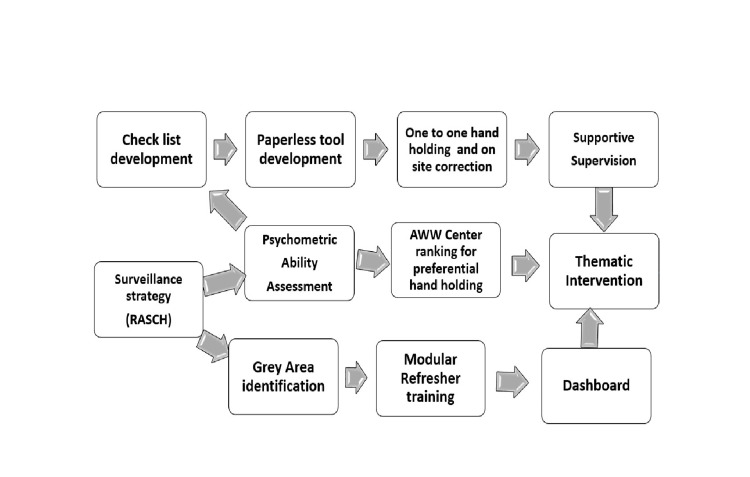
Intervention framework

The investigators have crafted the following intervention framework (Figure 5) based on insights received from this data-driven monitoring mechanism. We were able to identify the domain requiring more attention and develop a more focused monitoring strategy catering to the differential needs of the frontline workers. Based on this experience, we further propose exploring the psychometric analysis tools (such as Rasch scores) to identify specific activities to be focused on for an individual as per his or her ability. It also emphasizes consideration of the burden of given health problems in deciding monitoring and supervision plan. Thus, a data-driven framework proposed in this study will help prioritize monitoring and supervision of community-based management of acute malnutrition outreach sites. This strategy will ensure mentoring in identified individual-specific domains. The same framework can be suitably modified and adapted for monitoring and supervision of other public health programs.

## Conclusions

In summary, this strategy offers some distinct advantages compared to raw scored based ability estimation as discussed above. However, the effectiveness of the intervention framework will be achieved following the realistic application of focused monitoring and the improvement of the target indicators.

## Figures and Tables

**Figure 6:**
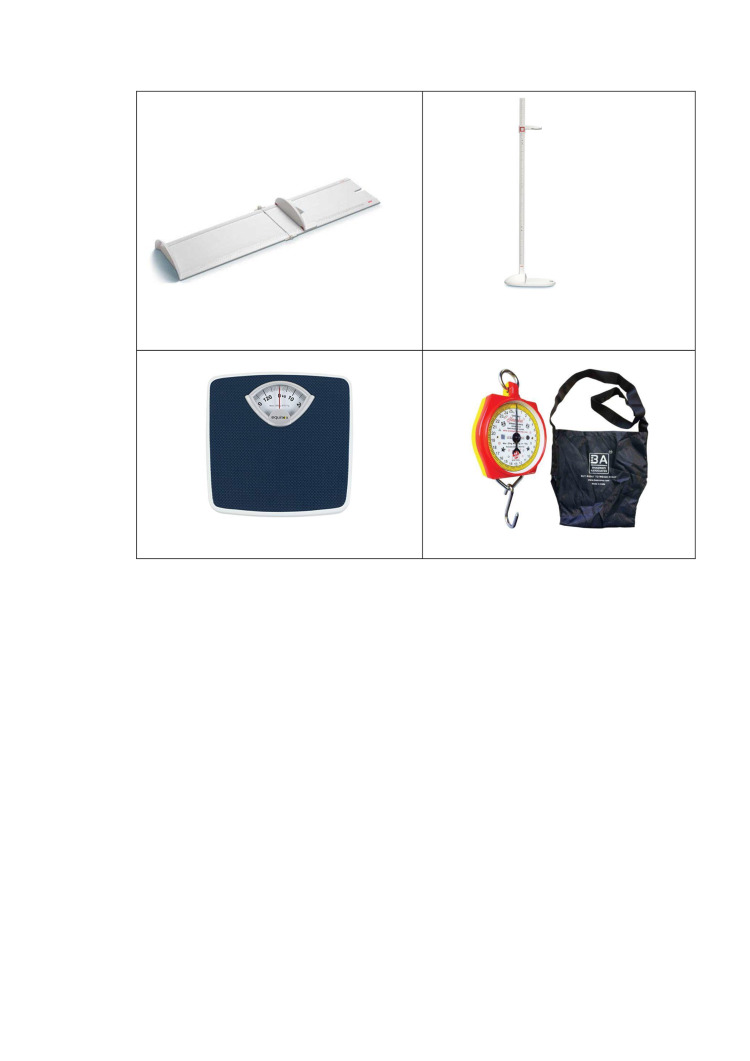
Image based response for Qn 6

**Figure 7:**
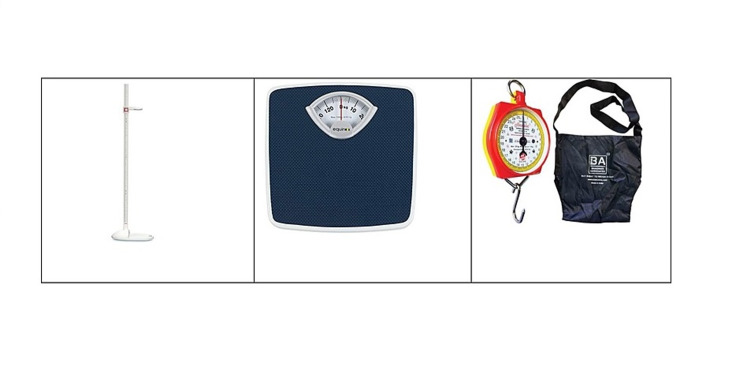
Image based response for Qn 7

**Figure 8:**
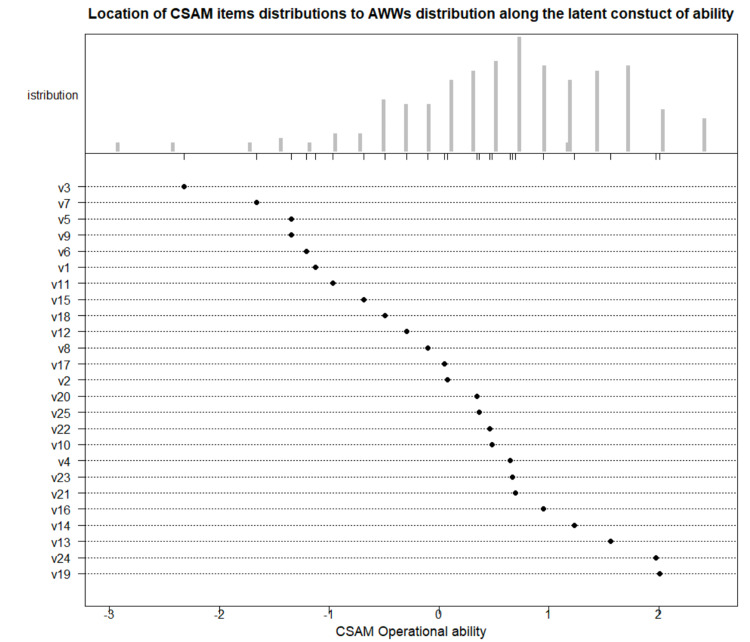
Location of CSAM items distribution to AWWs distribution along the latent construct of ability

## Appendices

Supplementary File 1: RASCH questionnaire

Exploring the capacity building areas employing RASCH analysis

AWW Centre No:

Village:

Name of the AWW:

Contact Number:

No of SAM Children registered at the centre in the current month/previous month:

Date of the interview:

1.      Who is at a maximum probability of  risk of falling under SAM category

a.      Laxmi, fourth girl child of a daily wage labourer

b.      Ravi, only male child of a school teacher

c.       Savitri, the  second girl child of a political leader

d.      Mukesh, a male child having a fever for 2 days

2.      A 10-month-old female child with 77 cm height and 6 Kg weight is having diarrhoea and is unable to drink or be breastfed. How will you respond?

a.      Give THR to the child

b.      Register the child under CSAM activity

c.       Refer the child to the NRC

3.      A 36-month-old normal male child is having severe diarrhoea. How will you respond?        

a.      Give ORS to the child

b.      Register the child under CSAM activity

c.       Refer the child to the NRC

d.      This child can be classified under MAM.

4.  Which is the correct order for measuring the height of a 26-month-old child?

a. let the child lie down on the board on his/her back, position head against the headboard. Knees and back straight, measure to the accuracy of 0.1 and note down the reading 

b. Remove their shoes, make the child stand against the stadiometer, Keep the knees together and Straight, Keep the child’s head straight, Bring the headpiece down onto the uppermost point on the child's Head, Record measurement to the last 0.1cm

c. Remove their shoes, make the child stand against the stadiometer, Keep the child’s head straight, Bring the headpiece down to the tip of the hair, Record measurement to the last 0.01cm.

5.  If a new child has come to Anganwadi Center during CSAM session, how will you confirm the age of the child.

a. By measuring the height of the child

b. By asking the mother

c. By checking the birth certificate

6. Which is the following depicted instruments will be used to measure the height of a 6-month-old child? (IMAGE ONLY)

7. Which is the instrument used to measure the weight of a 15-month-old child? (IMAGE only)

8. Which is the correct order for measuring the weight of an 18-month-old child?

a. Note the child’s age, Adjust the needle of the scale to zero, Put the child in the sling and hang  

it on to the hook, Make sure the child’s feet are not touching the ground, Read the weight 

when the child is calm and the needle stops moving

b. Note the child’s age, Adjust the needle of the scale to zero, Put the child in the sling and hang  

it on to the hook, Make sure the child’s feet are not touching the ground, hold on to the child 

for safety, Read the weight when the child is calm and the needle stops moving

c. Remove heavier clothes on the child, Adjust the needle of the scale to zero and make the child stand on the digital scale and note down the measurement.

9. If a family has a history of SAM and the second girl child (1 year old) from the same family is presented as normal, what will be your response?

a.      Refer the child to the NRC

b.       Will make frequent visits to the family

c.       Provide THR and monitor the feeding practices

10. If a child under the intervention hasn’t gained the desired weight in two months’ time, what will you do?

a. Follow up with the child every week

b. Follow up with the child every month

c. Provide more packets of THR

11. If the growth band of the child is in the yellow colour, what will you do?

a. Will register the child in the SAM category?

b. Will monitor the child and feeding practices

c. Will provide more packets of THR

12. Parents of an 18-month-old female registered child having uncomplicated SAM has been denying to feed the child with THR, How will you respond to this?

a. Report to the supervisor

b. Will visit the child’s family, listen to their issues and try to educate them on the need

c. Register the child in the SAM category

d. Immediate intervene for correcting measures in a forceful way by calling the family to the Aanganwadi centre

13. Which option is correct in the identification of bilateral pitting oedema

a. Press one foot of the child for 10 seconds and check for pitting

b. Apply thump pressure on the child’s feet for 3 seconds and check for pitting

c. Press both the feet of the child with both hands for 3 seconds and check for pitting

14.  Tick which option is incorrect if a child falls under the yellow or red zone in the growth chart

a. Inform the parents that their child's growth is slow for her/his age, Advise parents to pay greater attention to correct feeding practices and maintaining hygiene and cleanliness for their child.

b. Refer the child to the NRC

c. If the child is sick or weak, refer the child to the nearest NRC.

15. If a SAM child was staying at her mother’s home, which is your duty station and have shifted her residence to her father’s job location, How will you respond?

a. Report it to the supervisor

b. Report it to the NRC

c. Report it to the concerned Anganwadi at the child’s current residence.

16. What is incorrect about conducting the appetite test

a. The mother has to wash her hands before feeding the child

b. The child needs to be forced to complete the therapeutic food

c. It has to be made sure that the child was not fed for the last 2 hours.

17. Which of the following is a complicated SAM case

a. Child having Severe Dehydration

b. appetite pass

c. Child with edema in one foot

18. If you identify that the THR provided to a family is being shared among the whole family, how will you respond in this scenario (Tick the most appropriate)

a. Communicate to the parents regarding the need to feed THR to the child

b. Provide more packets of THR to the family as per family need

c. Will opt solely for monitored feeding of the child.

19. If a nearby AWW is unable to screen the child due to her incapacity and you came to know about this, how should you respond except-

a. Will communicate with the AWW and ask her to send the children to your centre for screening

b. Will do nothing as any intervention may create confusion at an administrative level.

c. Report it to the supervisor

d. will facilitate the help of ASHA of that village

20. Identify the child and categorize whether SAM, MAM or Normal.An  11-month-old male child with 60 cm height and 4.8 kg weight, No pitting edema but high fever

a.      SAM

b.      MAM

c.       Normal

21. Which of the following is correct regarding the THR supply?

a. 5 Packet of THR per week for Shalini who weighs 10.5 Kg

b. 2 Packet of THR per week for Aman who weighs 7 kg

c. 3 Packet of THR per week for Raziya who weighs 9 Kg

22. What is the frequency of follow up visits after discharge from CSAM intervention?

a. Once every week

b. Once every month

c. Once every fifteen days

23. If a child is under the CSAM intervention and you identify that the IFA and other medications are not given on correct dosage to the child. What will you do?

a. Teach the mother/ the caretaker about the correct dosage of the medicine

b. Supervised medication to ensure correct dosage

c. Report to supervisor

d All the above

24.  Please tick on the statement which is right-

a. wasting is determined in reference to age.

b. Stunting is determined in reference to height.

c. Wasting denotes the present/ short time nutrition status

d. Stunting denotes the present/ short time nutrition status.

25.  Which of the following is not true for CSAM program.

a. The target group for CSAM program is 6 -59 months children

b. CSAM session (satra) will be run at the sub-centre.

c. Registration will be done in the CSAM centre on VHSND in presence of ANM.

d. The risk of death by diseases like diarrhoea is more in SAM children compared to normal child

26. Home visit by AWW is intended to check the compliance for the following except-

a. THR diet

b. Amoxicillin and other drugs

c. Edema checking

Annexure-II

R-Code for RASCH Analysis

The datasheet consists of name of AWC,adjusted burden of SAM and MAM and response of 25 items(dicotomized )from 197 AWW.The RASCH analysis involes the erm package apart from base-r.

## Loading required package: MASS

##  Loading required package: msm

##  Loading required package: polycor

##

##  Attaching package: 'dplyr'

##  The following object is masked from 'package:MASS': ## ## select

##  The ## ##

##  The ## ##

following objects are masked from 'package:stats':

filter, lag following objects are masked from 'package:base':

intersect, setdiff, setequal, union

Rasch Priliminary modelling by using eRM package Item parameters for both eta(difficulty) and beta (easiness)

## ## Results of RM estimation: ## ## Call: RM(X = df1) ## ## Conditional log-likelihood: -2120.778 ## Number of iterations: 25 ## Number of parameters: 24 ## ## Item (Category) Difficulty Parameters (eta): with 0.95 CI:

## Estimate Std. Error lower CI upper CI ## v2 0.076 0.156 -0.230 0.382

## v3 ## v4 ## v5

-2.319 0.300 0.648 0.151 -1.344 0.212

-2.906 0.353 -1.761

-1.732 0.944 -0.928

## v6 ## v7 ## v8 ## v9 ## v10 ## v11 ## v12 ## v13 ## v14 ## v15 ## v16 ## v17 ## v18

-1.205 0.204 -1.659 0.235 -0.103 0.160 -1.344 0.212

0.486 0.151 -0.965 0.191 -0.292 0.164

1.560 0.160

1.234 0.154 -0.684 0.178 0.950 0.151 0.051 0.157 -0.495 0.171

-1.605 -2.120 -0.416 -1.761

0.189 -1.338 -0.614

1.246

0.932 -1.032 0.653 -0.256 -0.830

-0.805 -1.197 0.210 -0.928 0.782 -0.591 0.030 1.873 1.537 -0.335 1.247 0.358 -0.160

##  v19 2.009

##  v20 0.344

##  v21 0.695

##  v22 0.462

##  v23 0.672

##  v24 1.978

##  v25 0.368 ##

0.173 1.669 0.153 0.045 0.151 0.399 0.152 0.165 0.151 0.376 0.172 1.641 0.152 0.069

2.348 0.643 0.990 0.759 0.967 2.316 0.666

## Item Easiness Parameters (beta) with 0.95 CI:

##

##  beta v1

##  beta v2

##  beta v3

##  beta v4

##  beta v5

##  beta v6

##  beta v7

##  beta v8

##  beta v9

Estimate Std. 1.123

-0.076 2.319 -0.648 1.344 1.205 1.659 0.103 1.344

Error 0.199 0.156 0.300 0.151 0.212 0.204 0.235 0.160 0.212

lower CI 0.733 -0.382 1.732 -0.944 0.928 0.805 1.197 -0.210 0.928

upper CI 1.513 0.230 2.906 -0.353 1.761 1.605 2.120 0.416 1.761

## beta v10 ## beta v11 ## beta v12 ## beta v13 ## beta v14 ## beta v15 ## beta v16 ## beta v17 ## beta v18 ## beta v19 ## beta v20 ## beta v21 ## beta v22

-0.486 0.151 0.965 0.191 0.292 0.164

-1.560 0.160 -1.234 0.154 0.684 0.178 -0.950 0.151 -0.051 0.157 0.495 0.171 -2.009 0.173 -0.344 0.153 -0.695 0.151 -0.462 0.152

-0.782 0.591 -0.030 -1.873 -1.537 0.335 -1.247 -0.358 0.160 -2.348 -0.643 -0.990 -0.759

-0.189 1.338 0.614

-1.246 -0.932 1.032 -0.653 0.256 0.830 -1.669 -0.045 -0.399 -0.165

## beta v23 -0.672 0.151 -0.967 -0.376

## beta v24 -1.978 0.172 -2.316 -1.641 ## beta v25 -0.368 0.152 -0.666 -0.069

## [1] -1.123091

## beta v3 ## -2.32 ## beta v18 ## -0.49 ## beta v10 ## 0.49 ## beta v19 ## 2.01

beta v7 beta v5 beta v9 beta v6 beta v1 beta v11 beta v15 -1.66 -1.34 -1.34 -1.20 -1.12 -0.96 -0.68 beta v12 beta v8 beta v17 beta v2 beta v20 beta v25 beta v22 -0.29 -0.10 0.05 0.08 0.34 0.37 0.46 beta v4 beta v23 beta v21 beta v16 beta v14 beta v13 beta v24 0.65 0.67 0.69 0.95 1.23 1.56 1.98

person-item map

A person-item map displays the location of item (and threshold) parameters as well as the distribution of person parameters along the latent dimension. Person-item maps are useful to compare the range and position of the item measure distribution (lower panel) to the range and position of the person measure distribution (upper panel).Items should ideally be located along the whole scale to meaningfully measure the ‘ability’ of all persons.

plotPImap(res_rm_1, item.subset = "all", sorted = TRUE, main = "Location of CSAM items distributions to AWWs distribution

along the latent constuct of ability", latdim = "CSAM Operational ability",

pplabel = "AWWsPerson Paramter Distribution", cex.gen = 1, xrange = c(-3,2.5), warn.ord = TRUE, warn.ord.colour = "blue",irug = TRUE, pp = NULL)->pi_map (Figure- 8)

##Model Identificationidentification constraint, ##the identification constraint in RM() is a sum-to-zero constraint round(sum(betas), 10)

## [1] 0

Rasch diagnotics

Andersen’s Likelihood ratio test

test_data<-df1 test_data<-df1 model <- RM(test_data) LR_median <- LRtest(model, splitcr = "median") LR_median

##

## Andersen LR-test: ## LR-value: 34.315

## Chi-square df: 24 ## p-value: 0.079

#Andersen LR-test: # LR-value: 34.315 #Chi-square df: 24 #p-value: 0.079

####wald test chek for single items wt<-Waldtest(model) wt #q19, 24 and 25 have the large z value #after looking the construc there seems to be some typographical errors, in option b of q19 # q24 goes well with construct and will be included in final analyis #Question 25 mismatch with the construct and ‘satra’ and ‘up swathya kendra’ words needed to be look for.

# #those with large z-values in the Wald test . Do those items have special features that set them apart from the others in favoring one split group over the other?
